# Immunotherapy of WAP-T_NP_ mice with early stage mammary gland tumors

**DOI:** 10.18632/oncotarget.18850

**Published:** 2017-06-29

**Authors:** Michael Bruns, Wolfgang Deppert

**Affiliations:** ^1^ Heinrich-Pette-Institute, Leibniz-Institute for Experimental Virology, 20251 Hamburg, Germany; ^2^ Institute for Tumor Biology, University Medical Center Hamburg-Eppendorf (UKE), University of Hamburg, 20246 Hamburg, Germany

**Keywords:** transgenic breast cancer mouse model, SV40 T-antigen, LCMV NP T-cell epitope, adoptive transfer of low/high avidity CTLs, CTL exhaustion

## Abstract

The SV40 transgenic BALB/c mouse based WAP-T/WAP-T_NP_ model for triple-negative breast cancer allows the analysis of parameters influencing immunotherapeutic approaches. Except for WAP-T_NP_ tumors expressing the immune-dominant LCMV NP-epitope within SV40 T-antigen (T-Ag_NP_) which is not expressed by T-Ag of WAP-T tumors, the tumors are extremely similar. Comparative anti-PD1/PD-L1 immunotherapy of WAP-T and WAP-T_NP_ mice supported the hypothesis that the immunogenicity of tumor antigen T-cell epitopes strongly influences the success of immune checkpoint blockade therapy, with highly immunogenic T-cell epitopes favoring rapid CTL exhaustion. Here we analyzed the immune response in NP8 mice during early times of tumor development. LCMV infection of lactating NP8 mice induced lifelong tumor protection by memory CTLs. Immunization with LCMV after involution and appearance of T-Ag_NP_ expressing parity-induced tumor progenitor cells could not cure the mice, as memory CTLs became exhausted. However, immunization significantly prolonged the time of tumor outgrowth. Elimination of exhausted CTLs and of immunosuppressive cells by sub-lethal γ-irradiation, followed by adoptive transfer of NP-epitope specific CTLs into NP8 tumor mice with early lesions, completely prevented tumor outgrowth, when lymphocytes obtained after injection of weakly immunogenic NP8 tumor-derived cells into BALB/c mice were transferred. Transfer of lymphocytes obtained after infection of BALB/c mice with highly immunogenic LCMV into such mice delayed tumor outgrowth for a significant period, but could not prevent it. We conclude that eliminating exhausted CTLs and immune-suppressive cells followed by transfer or generation of low-avidity tumor antigen-specific CTLs might be a promising approach for curative tumor immunotherapy.

## INTRODUCTION

Defeating cancer by the body’s defenses, i.e. by inducing or fostering an immune response against tumor cells, has been the vision of cancer researchers since a long time. During the last years, important progress has been achieved with promise to make this dream come true. Current immunotherapeutic strategies comprise various approaches, including application of immune-modulatory drugs, cancer vaccines, oncolytic viruses, adoptive transfer of *ex vivo* activated autologous T-cells (naïve or genetically modified) and natural killer cells, application of antibodies against tumor-specific antigens (naïve or genetically modified), as well as various schemes of immune checkpoint blockade therapy (for a recent review see Farkona et al., 2016 [1]). However, despite all progress, success rates are still low and most immunotherapeutic approaches are still confined to clinical trials. An important hurdle in improving immunotherapeutic regimens is the difficulty to systematically analyze parameters influencing the outcome of such therapies in human patients, due to both, the inherent genetic diversity of patients and their tumors, as well as the limits on experimentation due to ethical considerations.

One way of systematically addressing at least some parameters influencing immunotherapy is the use of suitable animal systems. For this purpose, our laboratory has developed the cross-species validated transgenic BALB/c mouse based WAP-T models for triple negative breast cancer. For immunological studies, we use two different lines of tumor mice (WAP-T and WAP-T_NP_ mice), immunologically differing only in the expression of a single T-cell epitope in their major tumor antigen: WAP-T and WAP-T_NP_ mice contain both as transgene the Simian virus 40 (SV40) early gene region under control of the whey acidic protein (WAP) promoter. Upon transgene induction after parturition SV40 early proteins are expressed, with T-antigen (T-Ag) being the major tumor antigen. In WAP-T_NP_ mice, the SV40 transgene additionally encodes a highly immunogenic T-cell epitope, the NP_118-126_-epitope within the nucleoprotein (NP) of lymphocytic choriomeningitis virus (LCMV). While SV40 T-Ag expressed in WAP-T tumor mice is only weakly immunogenic in the BALB/c mouse background, the chimeric T-Ag/NP protein (T-Ag_NP_) in WAP-T_NP_ tumor mice is highly immunogenic. Except for this immunological difference, WAP-T and WAP-T_NP_ tumors are histologically and molecularly extremely similar.

Transgene induction, tumor growth, as well as histological and molecular tumor characteristics have been summarized recently in Bruns et al., 2015 [2] and 2016 [3]. In brief, after induction of the WAP-promoter, SV40 early proteins T-Ag, small t, and 17kT are expressed in epithelial cells of the mammary glands, with a peak during lactation around day 7 post partum (pp). Most T-Ag expressing cells are eliminated during involution, but parity-induced tumor progenitor cells with hormone-independent T-Ag expression appear about 10-14 days post weaning (pw) and give rise to hyperplastic lesions (days 20-40 pw). From then on these lesions develop into intraepithelial neoplasia (MIN) between days 60-90 pw in all terminal end-buds, from which only a few (2 to 6 per tumor mouse) develop into invasive carcinomas. On average, invasive tumors reach a size of 1.5 cm in diameter (experimental endpoint) after 107 days. However, tumors do not develop synchronously and exhibit different growth kinetics [4], resulting in a rather broad time span for tumors reaching endpoint size in individual mice (see also Figure 5 and Supplementary Table 1). We used the transgenic WAP-T_NP_ line NP8 and the WAP-T line T1, either containing the BALB/c mouse specific cytotoxic T lymphocyte (CTL) NP-epitope of LCMV within SV40 T-Ag (T-Ag_NP_ in NP8) or not (T-Ag in T1). Tumor development and tumor characteristics are similar for T1 and NP8 mice [2, 3].

Immunization of NP8 tumor mice with LCMV led to transient tumor regression, due to the presence of exhausted, programmed death-1 protein (PD1)-expressing NP-epitope specific CTLs. CTL activity could be largely restored by treatment of the mice with anti-PD1 antibodies [2]. Following up this observation we subjected NP8 and T1 tumor mice to immune checkpoint blockade therapy with antibodies against PD1 or its ligand PD-L1 [3]. While NP8 tumors had fully re-appeared already less than 14 days after therapy, T1 tumor mice showed a significantly prolonged period of tumor regression up to 31 days. Due to the close similarities of WAP-T and WAP-T_NP_ tumors, this difference could only be ascribed to the presence or absence of the NP-epitope in WAP-T_NP_ and WAP-T tumors, respectively. Indeed, we were able to show that the NP-epitope elicited a fast and strong epitope-specific CTL response, but at the same time also promoted rapid CD8^+^ T-cell exhaustion. On the other hand, the relatively good efficacy of the anti-PD1/PD-L1 treatment in WAP-T tumor mice supported the idea that tumors expressing weak tumor antigen T-cell epitopes respond much better to immune checkpoint blockade therapy because re-establishing an exhausted status of CTLs against these epitopes will take much longer [3].

We envisioned that the idea of “weak beats strong” regarding immunogenicity of T-cell epitopes in cancer immunotherapy [5] could be further exploited in combining immunization strategies with immune checkpoint blockade therapy. LCMV infection of NP8 tumor mice led to only transient tumor regression, and tumors re-grew already within the next three weeks [2]. However, treatment of NP8 tumor mice usually had been performed at time points where the mice contained invasive mammary carcinomas with diameters between 1 and 1.5 cm, which is huge in comparison to the size of a mouse. In the experiments described here, we systematically analyzed the NP-epitope specific immune response of NP8 mice early during tumor development with the aim to find conditions which might possibly allow a curative immunotherapy at this tumor stage. We show that endogenous NP-epitope specific memory CTLs in NP8 tumor mice become exhausted very early pw, when parity-induced tumor progenitor cells appear and form hyperplastic lesions [6]. Immunization with LCMV at these early stages of tumor development significantly prolongs the time for tumor outgrowth, but does not cure the mice. Curative immunotherapy, however, was possible when exhausted CTLs and immunosuppressive cells were removed by sub-lethal γ-irradiation, followed by adoptive transfer of low avidity CTLs obtained from BALB/c mice after injection of H8N8 cells, a NP8-tumor derived tumor stem cell line [7]. Treatment with anti-PD1/PD-L1 antibodies followed by transfer of H8N8-specific CTLs was less effective, but demonstrated the feasibility of combining adoptive, tumor-specific CTL transfer with immune checkpoint blockade therapy for an effective and possibly curative immunotherapy.

## RESULTS

### Treatment of NP8 mice at the peak of T-Ag_NP_ expression during lactation

**Figure 1:**
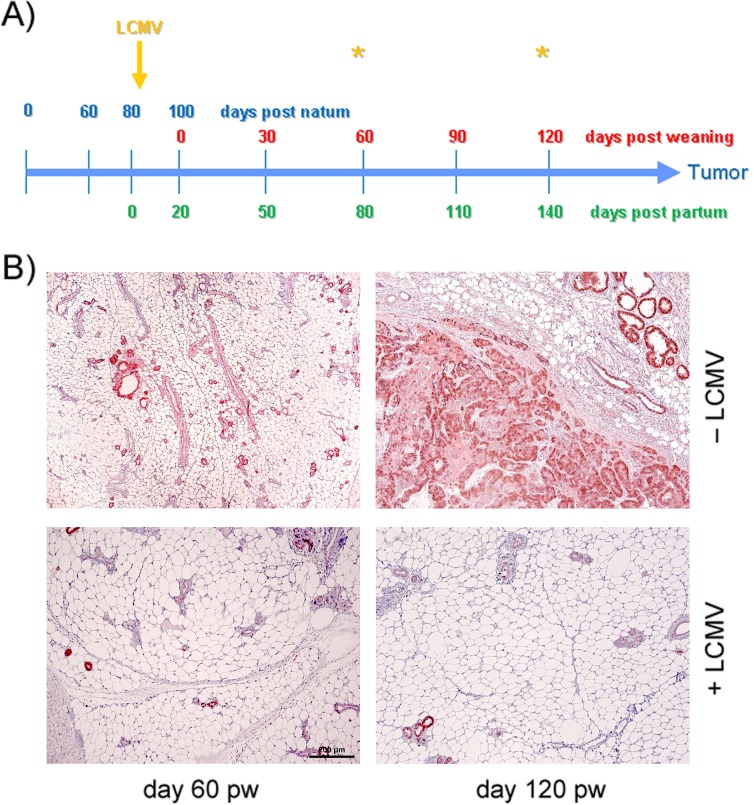
Analyses of mammary glands at days 60 and 120 pw **(A)** Time-line for acute LCMV infection of NP8 mice at day 7 pp. **(B)** Observation at different time points after infections; the upper panels represent the tissues of control mice, the lower panels those of infected mice. Bar, 200 μm.

In NP8 mice, expression of T-Ag_NP_ is induced during late pregnancy and reaches its peak during lactation on day 7 pp. We previously showed that LCMV infection or adoptive transfer of NP epitope-specific CTLs eliminates T-Ag_NP_ expressing cells in lactating mammary glands of NP8 mice [2]. Tumors in NP8 mice do not directly evolve from T-Ag_NP_ expressing cells in lactating mammary glands, but from parity-induced tumor progenitor cells arising after involution [6]. Therefore, we first analyzed, whether immunization on day 7 pp could prevent tumor outgrowth. Treated mice were histologically examined for tumor formation at days 60 and 120 pw (Figure 1A), i.e. at times when untreated NP8 mice had already developed early malignant lesions including MIN (day 60 pw) or had reached endpoint size (day 100-120 pw). Figure 1B, lower panel, reveals that LCMV immunization of NP8 mice at day 7 pp prevented tumor outgrowth completely. Even after extending the observation period up to 200 days pw revealed only very few T-Ag_NP_ positive ducts, which, however, failed to form hyperplastic lesions (Supplementary Figure 1, lower panels). In contrast, tumors in untreated mice had reached the endpoint size of 1.5 cm in diameter on average at day 107 pw (Figure 1B, Supplementary Figure 1, upper panels).

### Tumor outgrowth in day 7pp treated NP8 mice is controlled by CD8^+^ memory T-cells

**Figure 2:**
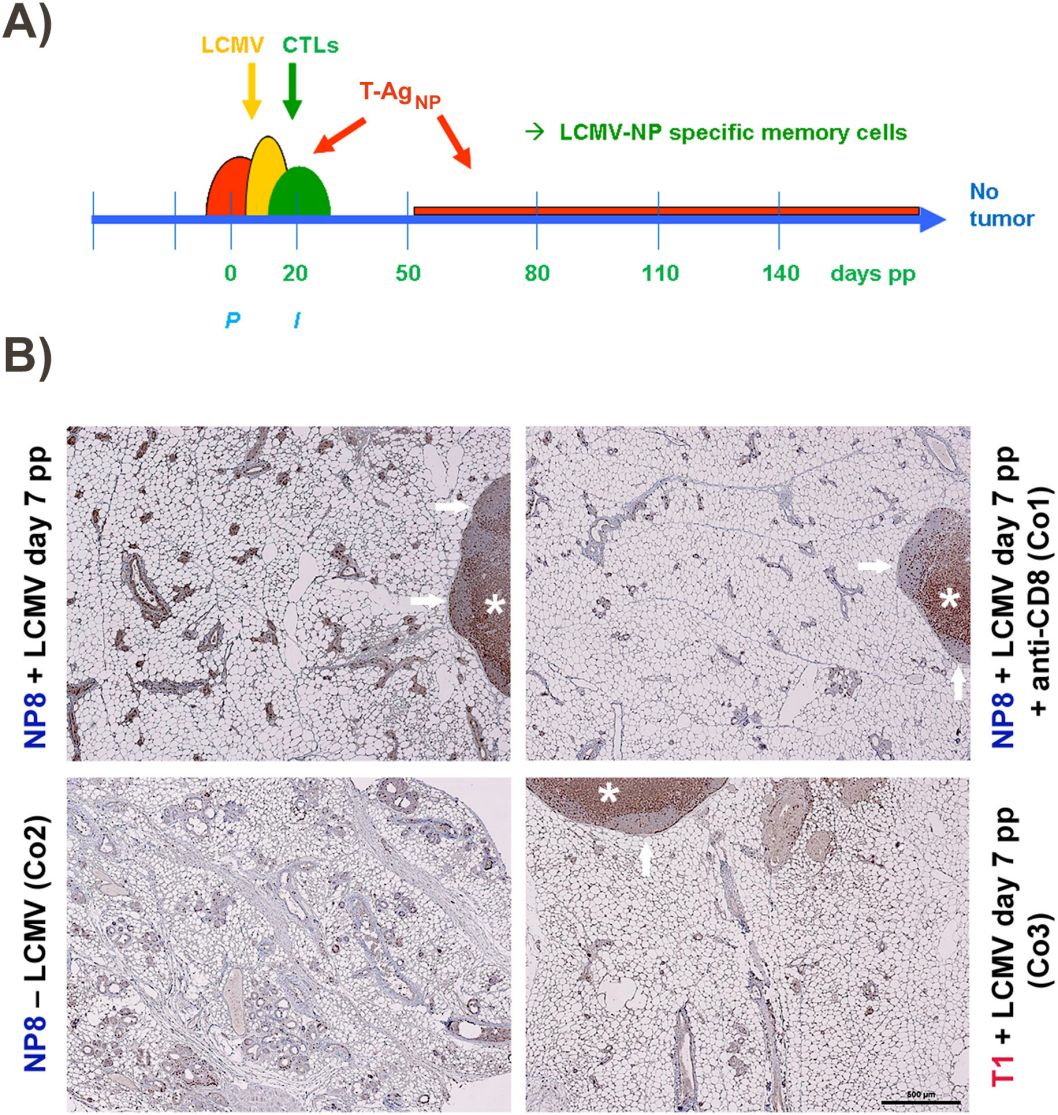
**(A)** Proposed mode of protection of NP8 mice against tumor development by infection with LCMV at day 7 pp. The semi-ovals in red, yellow, and green, represent the consecutively slightly retarded increases and decreases of SV40 T-Ag_NP_ production, LCMV infection, and LCMV NP-epitope specific CTL activation; *P*, partum, *I*, begin of involution. **(B)** Enrichment of memory CTLs within mammary glands of NP8 mice 120 days pw, after infection with LCMV at day 7 pp. The lymph nodes seen here partially served as internal controls, where CD3^+^ cells could also be localized in two of the three control experiments (e.g. Co1 and Co3) within the paracortex (*), whereas (as expected) no T cells could be visualized in the B cell areas of the secondary follicles (arrows). Bar, 500 μm.

Figure 2A proposes a model to explain the tumor-free state of NP8 mice treated with LCMV on day 7 pp. This model is based on the well-known immunology of lytic LCMV infection of BALB/c mice [8, 9]. Infection of NP8 mice with LCMV on day 7pp is followed by generation of NP-epitope specific CTLs which eliminate the virus and T-Ag_NP_ expressing cells within 14 days, thus reflecting the immune response of BALB/c mice acutely infected with LCMV [8, 9]. During acute infection, the number of activated CTLs rapidly decreases after virus elimination, with the exception of a few memory cells leading to life-long protection of the animals against re-infection [10]. We propose that day 7 pp immunized mice might be protected against appearance of tumor cells in a similar fashion, and argued that memory CTLs might be the main players (Figure 2A).

**Figure 3:**
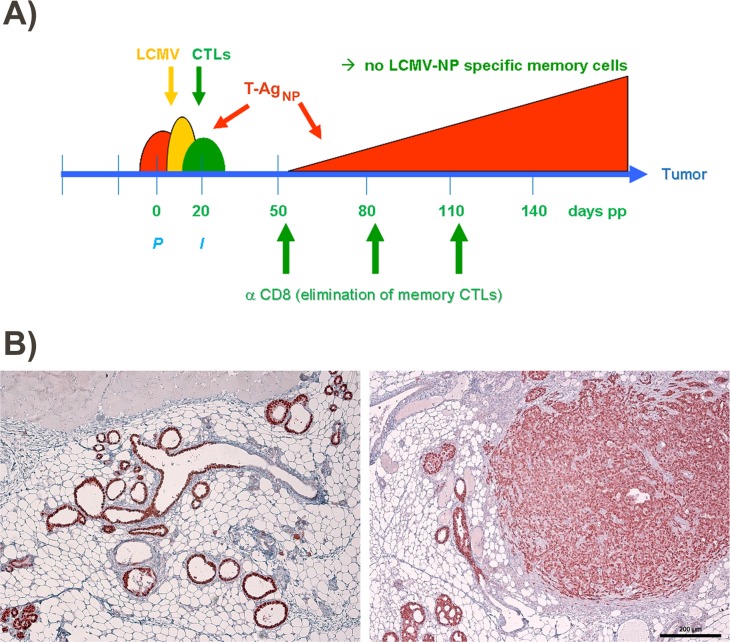
Scheme for treatment of LCMV infected NP8 tumor mice with anti-CD8 antibodies **(A)** To prove that memory CTLs are responsible for the healthy state of NP8 mice infected with LCMV at day 7 pp, the mice received anti-CD8 antibodies at days 50, 80, and 110, respectively, for the deletion of LCMV-NP specific CTLs. **(B)** T-Ag positive hyperplastic lesions in a mammary gland not carrying a tumor (left panel) and mammary carcinomas in various stages (right panel) from treated animals harvested and prepared 150 days pw. Bar, 200 μm.

To test this hypothesis, we first analyzed immunized NP8 mice for the presence of CD8^+^ memory T-cells. Histological analysis of mammary gland tissues performed 120 days pw showed an enrichment of CD8^+^ T-cells only in NP8 mice infected at day 7 pp. T-cells within the mammary gland tissues were detected using anti-CD3 antibodies (Figure 2B, left panel above). These cells were completely eliminated, when NP8 mice were treated with anti-CD8 antibodies, thus revealing their true nature as CD8^+^ T-cells (Figure 2B, right above, Co1). When the same experiment was performed with T1 mice, which express T-Ag, but not the NP-epitope [2, 3], no enrichment of CD3^+^ cells could be detected (Figure 2B, right below, Co3). Similarly, no CD3^+^ cells could be observed in uninfected NP8 mice (Figure 2B, left below, Co2). Next, we treated NP8 mice, immunized with LCMV on day 7 pp, with anti-CD8 antibodies three times (at intervals of 30 days) for the removal of all CD8^+^ cells and thus also memory CTLs (Figure 3A). Figure 3B shows that removal of memory CTLs from immunized mice led to tumor outgrowth.

### Treatment of NP8 mice 60 days post weaning

**Figure 4:**
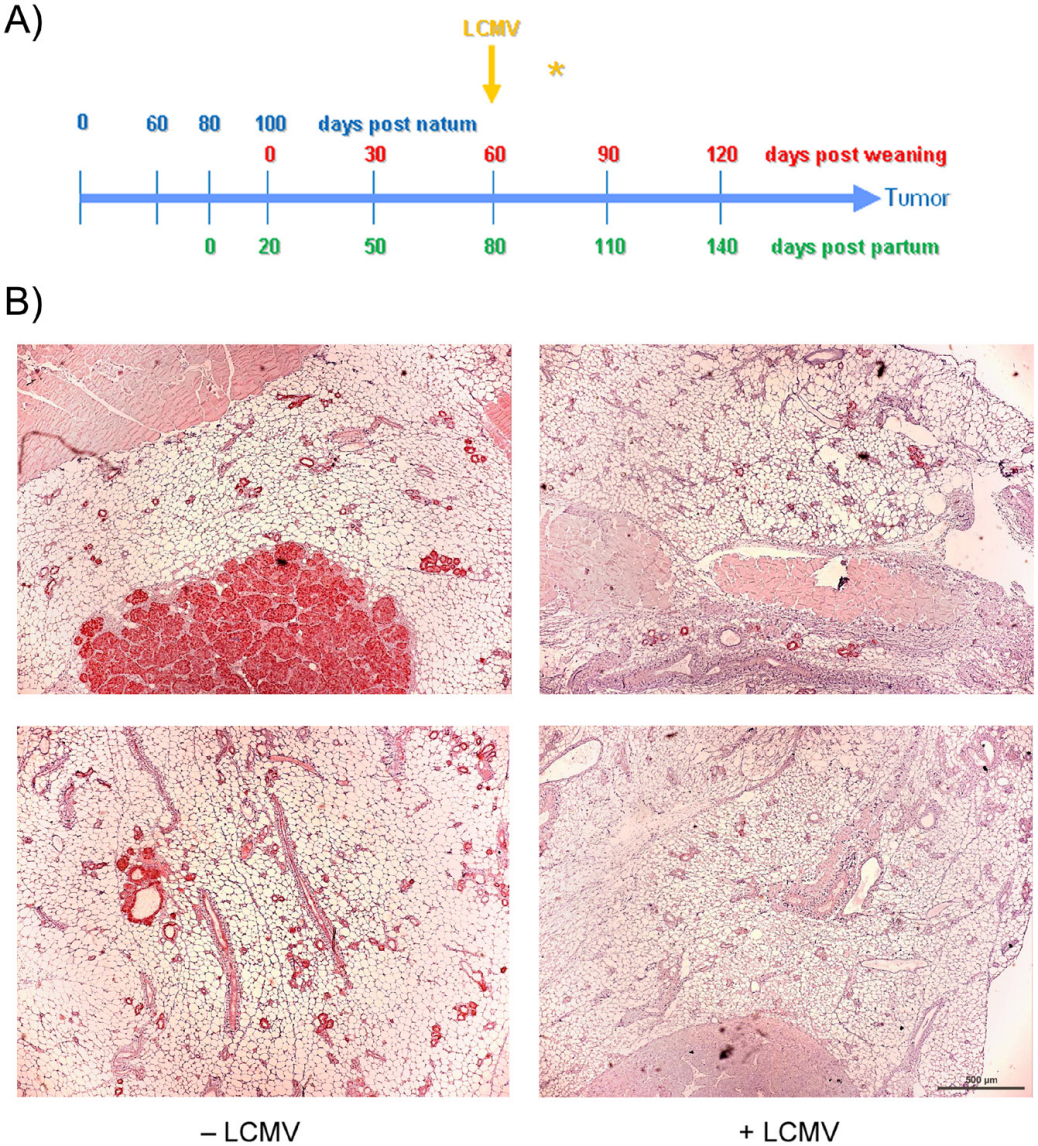
Therapeutic effect of LCMV immunization starting early in tumor growth **(A)** scheme; **(B)** immune-histochemistry of T-Ag. NP8 mice were infected at day 60 pw and analyzed 14 days later (right panel); uninfected animals served as negative controls (left panel). Bar, 500 μm.

**Figure 5:**
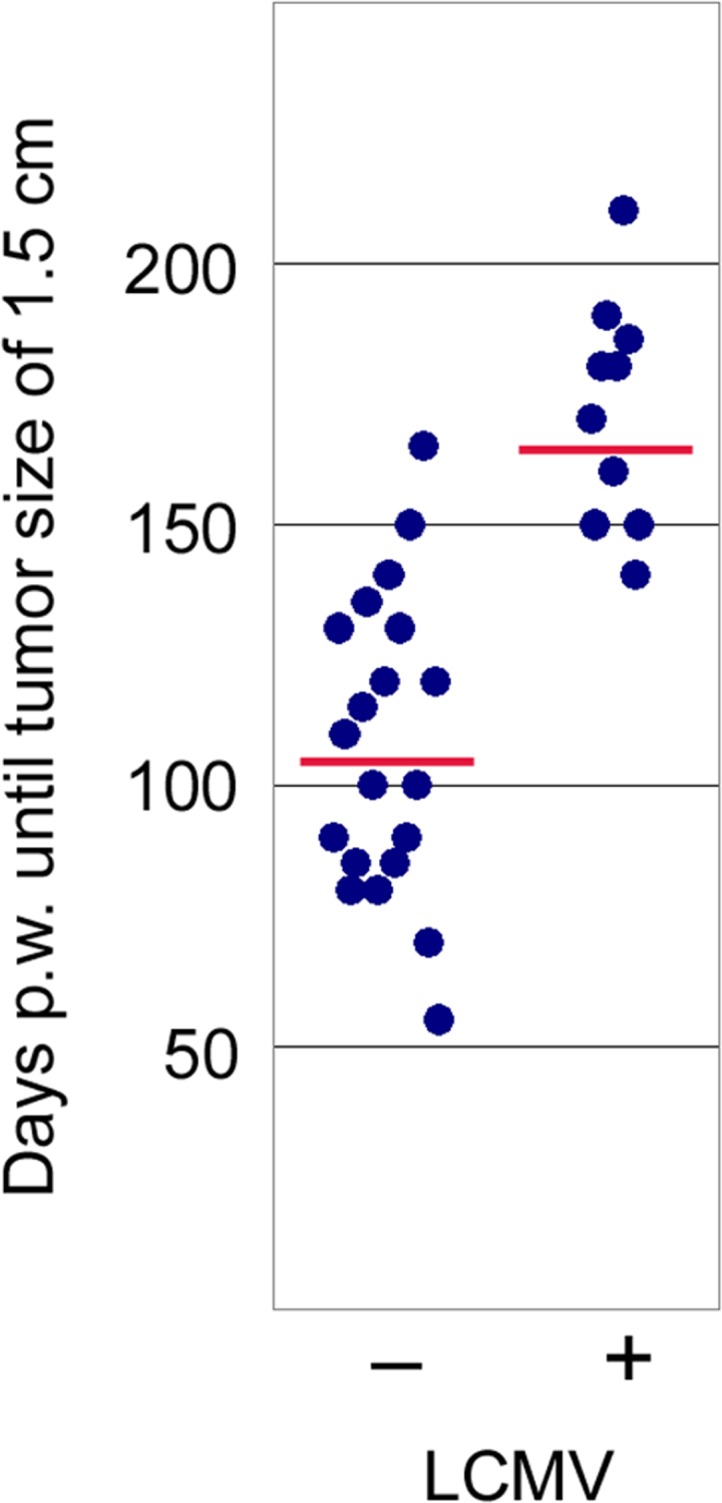
Comparison of tumor development in NP8 mice infected at day 60 pw [right dots, n = 10; means = 172 days, with a 95% confidence that the population mean (μ) falls between 156 and 187 days] with that of uninfected NP8 mice [left dots, n = 20; means = 107 days, with a 95% confidence that μ falls between 94 and 120 days]; y-axis shows time until tumors had grown to a size of 1.5 cm in diameter; p-value_infection vs control_ < 0.00001 significance at p < 0.01

We next analyzed whether LCMV infection induces tumor protecting CD8^+^ memory T-cells also at a rather early stage of tumor development. NP8 mice were infected at day 60 pw (Figure 4A, yellow arrow), when tumors mostly consisted of hyperplastic lesions and sporadic MIN; analyses were performed 14 days later. It is apparent that tumor development in LCMV treated mice had not significantly progressed during this period (Figure 4B, right panels) in comparison to untreated mice (Figure 4B, left panels). However, also this treatment scheme only prolonged the time for tumor outgrowth and thus did not cure the animals: while treatment of mice with fully developed tumors led only to a transient tumor regression period of 21 days [2], the early treatment applied here extended the average period of tumor growth to endpoint size (Ø 1.5 cm) by 65 days compared to untreated mice (Figure 5). Thus treatment at an early stage of tumor development protected animals from tumor outgrowth over a statistically significant period. Interestingly, time spans until tumors had reached endpoint size in LCMV treated versus untreated NP8 mice (Figure 5) showed a much higher variation in individual time spans than that of treated ones. The reason behind is unknown to us.

### CTLs in NP8 mice with early stage tumors (60 days pw) are exhausted

**Figure 6:**
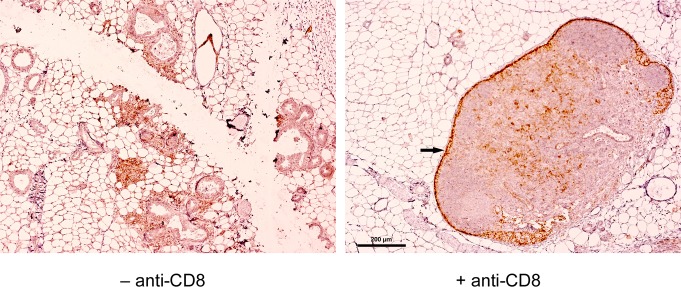
Presence of PD-L1+ lymphocytes in the mammary glands of NP8 mice when infected at day 60 pw, which were eliminated in mice treated before with anti-CD8 antibodies; the dark staining of cells within the capsules of lymph nodes (arrow on the right picture) was caused by an abundant presence of macrophages (Wanger et al. unpublished), which are not removable by anti-CD8 antibodies and therefore served as an internal control In addition, other PD-L1^+^ cells, which could represent further macrophages, T helper cells, NK cells, or regulatory T cells, are visible inside of the lymph nodes. Bar, 200 μm.

In NP8 tumor mice CTL exhaustion plays a decisive role for the inability to eliminate cancerous cells over a longer period [2, 3]. We therefore reckoned that CTLs might become exhausted already at an early stage of tumor development. We previously showed that anti-PD-L1 treatment of NP8 tumor mice efficiently eliminated exhausted CTLs, most likely due to enhanced expression of PD-L1 [3]. Indeed, PD-L1^+^ lymphocytes could be detected in NP8 mice infected with LCMV at day 60 pw in and around tubuli and early malignant lesions by staining mammary gland tissue with anti-PD-L1 antibodies 14 days after infection (Figure 6, left panel). When such NP8 mice had been treated before with anti-CD8 antibodies, their mammary glands were devoid of PD-L1^+^ lymphocytes, supporting the view that these lymphocytes represented exhausted CTLs. On the other hand, PD-L1^+^ macrophages, concentrating within the subcapsular sinus of lymph nodes could still be detected as they cannot be removed by anti-CD8 antibody treatment (Figure 6, right panel; Wanger et al., in preparation).

### Curative immunotherapy of NP8 tumor mice with early stage tumors

**Figure 7:**
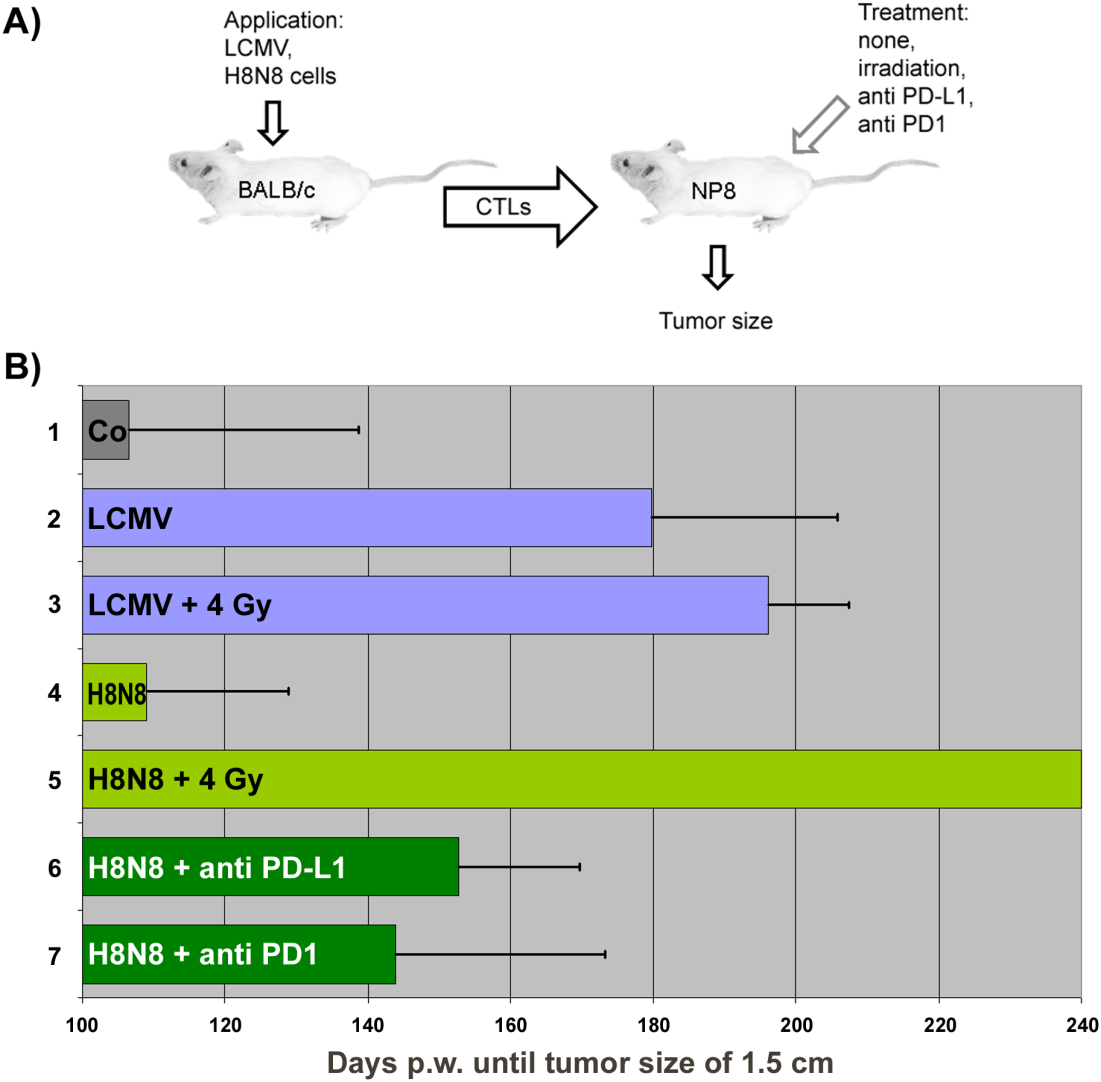
Therapeutic efficiencies after different immunologic treatments of NP8 tumor mice **(A)** Experimental outline of experiments for curative immunotherapy of NP8 mice with early stage tumors. **(B)** Adoptive transfer of differently generated NP-epitope specific CTLs to differently pretreated NP8 mice during an early time of breast cancer development and their influence on tumor outgrowth until tumors reached a size of about 1.5 cm; in brackets p-values_treatment vs control_ for significance: 1) Untreated NP8 mice, 2) Transfer of LCMV activated CTLs to NP8 mice [p = 0.000036 at p < 0.01], 3) Transfer of LCMV activated CTLs to irradiated NP8 mice [p < 0.00001 at p < 0.01], 4) Transfer of H8N8 activated CTLs to NP8 mice [p = 0.913833; not significant], 5) Transfer of H8N8 activated CTLs to irradiated NP8 mice [p < 0.00001 at p < 0.01], 6) Transfer of H8N8 activated CTLs to anti-PD-L1 treated NP8 mice [p = 0.002805 at p < 0.01], 7) Transfer of H8N8 activated CTLs to anti-PD1 treated NP8 mice [p = 0.09422 at p < 0.05].

The insufficient therapeutic efficiency of LCMV immunization of NP8 mice with early stage tumors is due to exhaustion of NP-epitope specific CD8^+^ T cells and possibly the presence of other immune-suppressive cells, e.g. regulatory T [11] and B [12] cells (T_reg_ and B_reg_). Therefore, we considered to improve our therapy scheme by eliminating exhausted CTLs and immune-suppressive cells before therapy. Sub-lethal γ-irradiation with 4 Gray (Gy) was chosen as the most efficient treatment option, as it eliminates, amongst other immune cells, all T cells, including (exhausted) CD8^+^ T-cells and T_reg_, as well as B_reg_ cells. Alternatively, we also applied PD-L1 treatment. For immunization, we opted for adoptive transfer of NP-epitope specific CTLs from immunized BALB/c mice out of two reasons: First, we previously found that CTLs derived from LCMV infected BALB/c mice had been very effective in clearing lactating mammary glands of NP8 mice from T-Ag_NP_ expressing cells [2]. Adoptive transfer thus might be more effective than direct immunization with LCMV. Second and most important, adoptive transfer also allowed us to immunize NP8 tumor mice with CTLs obtained from BALB/c mice after immunization with NP8 specific tumor cells, using the previously described H8N8 tumor stem cell line [7]. Thereby we created a setting that closely mimics transplantation of activated autologous tumor-specific CTLs in tumor therapy. Our experimental set-up is outlined in Figure 7A.

BALB/c mice served as donors of active NP-epitope specific CTLs, NP8 mice with early stage tumors as acceptors after various types of pre-treatment. Lymphocytes containing freshly generated CTLs from BALB/c mice were transferred to either untreated NP8 tumor mice or to tumor mice, in which exhausted CTLs as well immunosuppressive cells had been eliminated via a sub-lethal irradiation dose or, alternatively, by anti-PD1/PD-L1 antibody treatment. A detailed listing of the individual experiments and corresponding results is given in Supplementary Table 1 and is summarized in Figure 7B. Experiment 1 shows the control: tumors in untreated NP8 tumor mice reached the maximum tumor size of 1.5 cm in diameter (endpoint) on average 107 days pw (Figure 7B, lane 1, also shown in Figure 5, left). Transfer of LCMV specific CTLs into untreated NP8 tumor mice significantly increased the average time for endpoint tumor size to 180 days pw (Figure 7B, lane 2), and thus was more efficient (by 15 days) than LCMV infection (compare Figure 5). Interestingly, prior γ-irradiation of NP8 acceptor mice with 4 Gy irradiation enhanced the average time for tumor growth to 1.5 cm only slightly (Figure 7B, lane 3) and was not significant (p-value = 0.233951 at p < 0.1). This is reminiscent of our previous finding that an efficient immune response in PD-1/PD-L1 treated NP8 tumor mice led to rapid CTL exhaustion [3] and indicates that the high avidity CTLs induced by LCMV infection of BALB/c mice had become rapidly exhausted in the NP8 tumor mouse environment.

In contrast, transfer of CTLs induced by injection of H8N8 cells into BALB/c mice did not significantly increase the time of tumor growth to endpoint, demonstrating that these CTLs elicited only a rather weak NP specific immune response (Figure 7B, lane 4). This most likely reflects that the NP-epitope in H8N8 cells is only weakly immunogenic in BALB/c mice (for possible explanations see Discussion). As our previous data had shown that a weak immune response, and thus the generation of low avidity CTLs, prevents rapid CTL exhaustion [3, 5], we analyzed the effect of transfer of H8N8 specific CTLs into NP8 tumor mice irradiated with 4 Gy prior to transfer. Such treatment not only significantly prolonged the time for tumor outgrowth, but completely cured the animals (Figure 7B, lane 5). Single treated animals were kept up to 300 days without any signs of tumor development. Also pre-treatment of NP8 tumor mice with anti-PD1/PD-L1 antibodies before transfer of H8N8 specific CTLs significantly increased the time of tumor outgrowth compared to transfer of H8N8 specific CTLs alone, although it was less effective than pre-treatment of NP8 tumor mice with 4 Gy irradiation (Figure 7B, lanes 6, 7).

**Figure 8:**
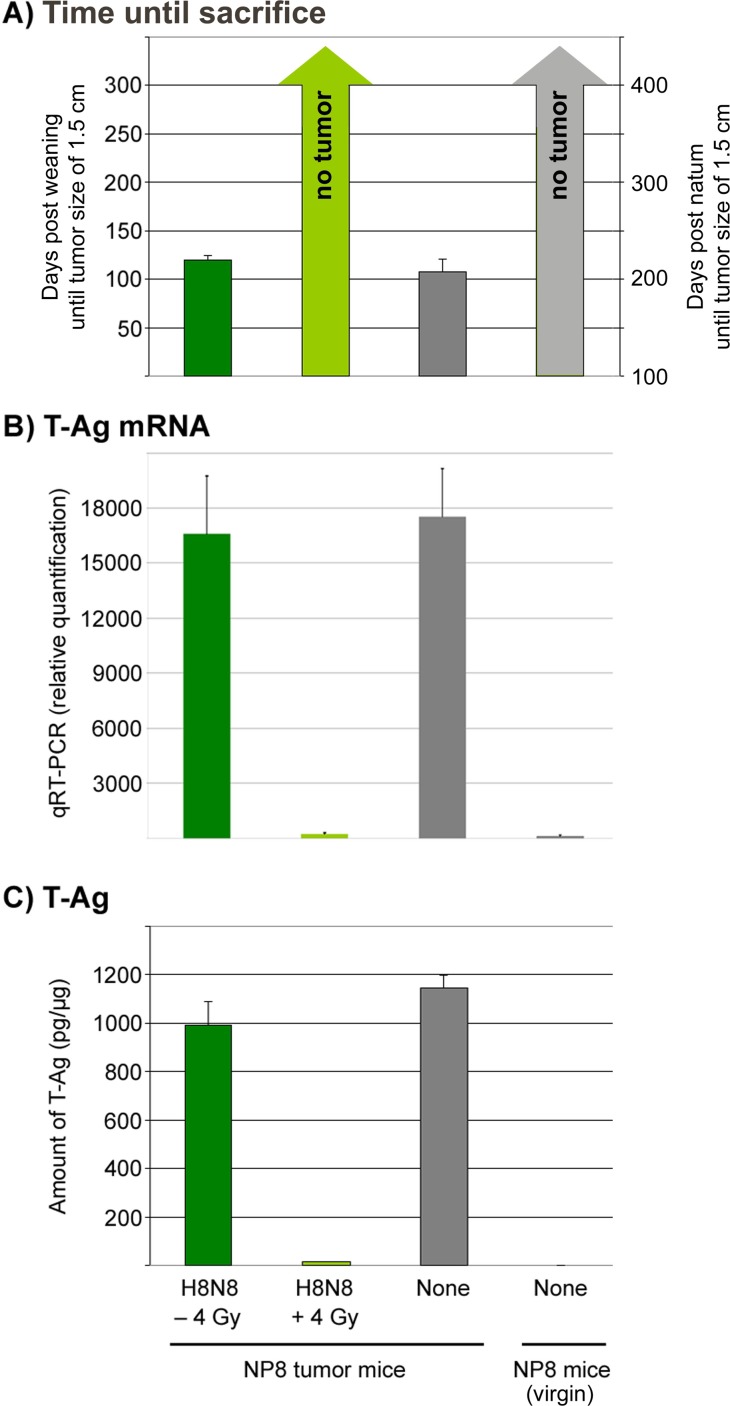
Quantification of mRNAs and proteins for T-Ag either in non-irradiated tumor mice treated with H8N8 cells (dark green columns) or in irradiated tumor mice treated with H8N8 cells (light green columns) in comparison to untreated tumor mice (dark grey columns) or untreated non-induced (virgin) NP8 mice (light grey columns) **(A)** Days post weaning (for tumor mice) or alternatively post natum (for virgin) mice until tumor sizes reached 1.5 cm or until day 300/400 of sacrifice. **(B)** Relative quantities of mRNA for T-Ag calculated by qRT-PCR. **(C)** Amount of pg T-Ag per μg of mammary gland tissues measured by ELISA.

To further substantiate the successful treatment of NP8 tumor mice by γ-irradiation followed by transfer of H8N8 specific lymphocytes, we analyzed mice 300 days pw (i.e. at an age of 400 days) for tumor free survival until sacrifice (Figure 8A), expression of T-Ag mRNA (Figure 8B) and T-Ag protein (Figure 8C). It is evident that γ-irradiation of NP8 mice followed by transfer of H8N8 specific CTLs completely blocked T-Ag expression and thus tumor development over the whole life span of NP8 mice.

## DISCUSSION

In this study we analyzed the effects of LCMV NP-epitope specific immunotherapy of NP8 mice at early stages of tumor development, as we hoped to achieve better responses than in tumor mice harboring large tumors. Indeed, induced NP8 mice, LCMV treated at the peak of T-Ag_NP_ expression during lactation (day 7 pp), were completely protected from tumor outgrowth by NP-specific memory T-cells that had developed after LCMV infection. This finding can be explained by assuming that the non-replicating, T-Ag_NP_ expressing mammary epithelial cells did not significantly influence the immune response to LCMV, and that at this stage the immune response of infected NP8 mice reflected that of lytic infection of BALB/c mice, which protects mice life-long against re-infection by LCMV [10]. Immunization of NP8 mice with LCMV at day 7 pp thus can be considered as tumor-protective.

Surprisingly, the immune status of induced NP8 mice changed dramatically already very early after weaning. As can be deduced from the data listed in Supplementary Table 1, immunization by adoptive transfer as early as 21 days pw could neither prevent tumor outgrowth, nor prolong the time of protection over that achieved by immunization at later times (i.e. 60 days pw), although 21 days pw only T-Ag_NP_ expressing cell clusters, local hyperplastic lesions and very few MIN can be detected in involuted mammary glands. However, in contrast to non-replicating T-Ag_NP_ expressing epithelial cells in lactating mammary glands, T-Ag_NP_ expressing cells in involuted mammary glands represent replicating parity-induced tumor progenitor cells arising after involution [6]. These cells probably stimulate both, generation of NP-epitope specific CTLs as well as their exhaustion, as also exemplified in Figure 6. In addition, such cells might already create an immune-protective microenvironment. This would imply that endogenous immune protection against tumor cells will become compromised once replicating tumor progenitor cells are present. Thus, intervention by immunization even at very early stages of tumor development usually can only provide a transient protection against tumor outgrowth, but will not be able to prevent it.

The most important finding of this study, however, is that at least for early stage malignancies in NP8 tumor development curative immunotherapy was nevertheless possible: removal of immune cells, specifically exhausted T-cells and immune-suppressive cells, by sub-lethal γ-irradiation, followed by adoptive transfer of CTLs obtained from BALB/c mice transplanted with H8N8 cells completely prevented tumor outgrowth up to 300 days pw (corresponding to 400 days total life time, and thus exceeding the usual life time of a laboratory mouse). Clearly, a major prerequisite for this positive result was the removal of exhausted immune as well as of immune-suppressive cells, but as important was the choice of CTLs for adoptive transfer. This is demonstrated by comparing the transfer of H8N8-specific CTLs with that of LCMV-specific CTLs, respectively, into NP8 tumor mice with or without prior irradiation of NP8 mice: While transfer of H8N8-specific CTLs into non-irradiated NP8 tumor mice did not significantly prolong the time for tumor outgrowth, transfer of LCMV-specific CTLs was very effective in prolonging this time. On the other hand, prior γ-irradiation did not significantly boost the effect of the transferred LCMV-specific CTLs. We interpret this result as further support of the pattern outlined in our previous study: weak immunogenic stimulation will result in a weak immune response, but will not induce CTL exhaustion, or at least delay it. Strong immunogenic stimulation will induce a strong immune response, but also induce rapid CTL exhaustion. This interpretation is in line with previous experimental data showing that upon contact with their targets high avidity CTLs become more easily eliminated or exhausted than low avidity CTLs [13, 14].

Considering that the major immunogenic T-cell epitope in H8N8 cells is the LCMV NP-epitope in T-Ag_NP_, which is also the major immunogenic T-cell epitope in LCMV infected BALB/c mice, the question arises why CTLs generated by injection of H8N8 cells into BALB/c mice elicit a rather weak immune response (i.e. are of low avidity) compared to high avidity CTLs derived from LCMV infected BALB/c mice. In addition to the different routes of immunization (replicative viral infection versus tumor cell injection), the simplest explanation is the difference in amount of NP-epitope presented by H8N8 cells compared to NP-epitope presentation during lytic infection. This interpretation corresponds well to our observation that H8N8 cells, despite presenting the NP-epitope, are able to transiently grow in BALB/c mice in a dose dependent manner (Supplementary Figure 2). As an additional support for the lower immunogenicity of the NP-epitope in H8N8 cells, transplantation of these cells into T1 mice, which express SV40 T-Ag, but not the NP-epitope [2], is successful in about 60% of the transplantations, while transplantation of H8N8(Arm) cells (i.e. H8N8 cells stably infected with the replication-defective Armstrong strain of LCMV and expressing large amounts of NP protein [3]; see also Materials and Methods) into T1 mice never led to tumor formation (Supplementary Table 2). We thus assume that the low avidity of CTLs induced by injection of H8N8 cells into BALB/c mice is the result of the weaker immunogenicity of the NP-epitope in this experimental setting. Their low avidity prevents their exhaustion and will allow the development of NP-specific memory T-cells that control tumor outgrowth. In addition, it is also possible that the lymphocytes transferred from H8N8 injected BALB/c mice contained CTLs directed against H8N8 specific tumor antigens other than the NP-epitope. One can assume that also these CTLs were of low avidity, as their transfer into NP8 tumor mice not pre-treated by γ-irradiation was virtually non-effective. However, it has been demonstrated that tumors expressing many mutated proteins respond significantly better to immune checkpoint blockade therapies [15].

An interesting aspect in the results of our curative treatment schemes is that anti-PD-L1 pre-treatment of the tumor mice was less efficient than pre-treatment by γ-irradiation, although anti-PD-L1 treatment in addition to exhausted CTLs should also eliminate PD-L1^+^ suppressive T_reg_ and B_reg_ cells [16]. Aside from technical aspects, like time of treatment before transfer, single or repeated treatments, etc., sensitization for anti-PD-L1 treatment might be a promising option. For example, Gong et al. 2017 [17] recently described that combined radiotherapy and anti-PD-L1 antibody treatment was able to synergistically enhance the anti-tumor effect in Non-Small Cell Lung Cancer (NSCLC), probably via PI3K/AKT and STAT3 pathways. In addition, PD-L1 expression could be enhanced before treatment by various chemokines, like e.g. TGF-Δ [18], as well as by induction of EMT [19, 20, 21].

Immune responses against tumors are very complex and clearly not restricted to CD8^+^ T-cells, as in the analyses described herein. However, at least for most SV40 induced mouse tumors CTLs in combination with CD4^+^ helper cells are the major players in an effective anti-tumor response in BALB/c [22, 23, 24] as well as in C57BL/6 mice [25, 26, 27]. In addition, as already outlined in our previous paper [2], NK cells might play a role in anti-tumor immunity against SV40 induced tumors [28].

Despite the reduced complexity of our approach, it might provide leads as how to progress towards regimens for curative immunotherapy of cancer. Transfer of activated autologous T-cells is in use in human cancer trials, and depletion of exhausted CTLs by immune checkpoint blockade therapy, too. Our data here show that already a single treatment of NP8 mice with anti-PD1/PD-L1 strongly enhanced the efficiency of CTLs derived from H8N8 injected BALB/c mice. Thus combining and refining these two approaches might be a promising path leading to successful cancer immunotherapy. We are, however, aware that early malignant lesions will not be the major area of application for such an immunotherapy, but rather the treatment of residual disease after conventional treatment of the primary tumor. It remains to be seen, whether such treatments can be successfully applied under these conditions. Our model system will allow to address this question in a preclinical setting using orthotopic transplantation of NP8 mouse specific H8N8 tumor stem cells, followed by primary tumor removal either by surgery [7] or by chemotherapy [4] before combinatory immune therapy.

## MATERIALS AND METHODS

### Mice

Inbred BALB/c mice were crossed with BALB/c-based transgenic mice and female transgenic offspring mice were selected for use in the experiments presented here. We used the transgenic WAP-T_NP_ (NP8) and WAP-T (T1) lines containing either the BALB/c mouse specific CTL NP-epitope of LCMV within the SV40 T-Ag (T-Ag_NP_ in NP8) or not (T-Ag in T1), respectively [2, 3]. Tumor development and tumor characteristics are similar for T1 and NP8 mice.

If not stated otherwise, at least three mice per group were used in each experiment. Statistic analyses were performed with the Single-Sample Confidence Interval Calculator and the One-way ANOVA Calculator programs. Mice were kept in S1 animal facilities, held under specific pathogen-free conditions and handled according to German regulations for animal experimentations. All protocols had been approved by the Hamburg administration (Deppert/Bruns #13/06; Deppert/Wanger #20/10: “Früherkennung der Entstehung von Mammakarzinomen für immuntherapeutische MaΔnahmen im transgenen Mausmodell”).

### Virus and cells

The triple plaque-purified WE strain of LCMV [29] was propagated and titrated in mouse NCTC clone 929 L cells using minimal essential medium supplemented with nonessential amino acids and 5% heated fetal calf serum (FCS) at 37°C [30]; the virus was quantitatively expressed as numbers of plaque-forming units (PFU) [31]. Transgenic mice were infected with 10^5^ PFU of LCMV intravenously (iv) or intraperitoneally (ip).

Cultures of tumor-derived G-2 or H8N8 cancer cells served in particular for the precise calculations of tumor growth during therapeutic treatments. The procedures for their isolation and their growth characteristics were described elsewhere [7, 32, 33, 34]. For the establishment of persistently LCMV-infected H8N8 cells we developed H8N8(Arm) cells by infection of H8N8 cells with the highly attenuated L(Arm) virus (mouse L cell-derived Armstrong strain of LCMV), followed by cultivation over 8 passages (16 days), similarly as described for the production of G-2(Arm) cells [3]. The cells were maintained in Dulbecco’s modified Eagle’s medium DMEM containing 10% FCS, 2 mmol/l glutamine and cultivated at 37°C in a humidified atmosphere of 5% CO_2_.

### Treatments of mice

For anti-PD-L1/PD1 treatment experiments we used goat polyclonal B7-H1/PD-L1 or PD1 antibodies (R&D Systems). The dose-dependency for clearance of the exhausted immune status using the antibodies in NP8 and T1 tumor mice was evaluated in previous analyses [3]. An iv dose of 0.5 mg of either antibody per kg mouse body weight was selected for the studies providing a strong protective reaction [3].

Adoptive transfer experiments were performed as already described [2]. Briefly, wt BALB/c mice were either infected with 10^2^ PFU of LCMV iv or were alternatively treated with 10^5^ H8N8 tumor cells ip. Prior to the transfer of immune cells from donor (BALB/c) mice, the acceptor (NP8 tumor) mice were γ-irradiated sub-lethally with a radiation dose of 4 Gy by the Cs-137 source (2,500–3,000 Curie) of a LISA I apparatus (Conservatome) to remove exhausted CTLs for about two to three weeks (personal experience). A similar extensive discharge of CTLs was obtained in mice, when they were treated with 400 μg of monoclonal anti-CD8 antibodies (mAbs) iv [35, 36]. In any case no impairment of health or variation in physiological behavior could be observed in mice undergoing such treatments.

### Transplantation of tumor cells

For transplantation experiments 1×10^2^ to 1×10^5^ tumor cells were harvested from cultures and re-suspended in 50 μl of a 1:1 mixture of serum-free DMEM and BD Matrigel Matrix high concentration, growth factor reduced (BD Bioscience). Between 10 and 20 weeks old non-induced (virgin) female mice were anaesthetized by ip injection of ketamine/xylazine. After an incision of about 5 mm into the skin the cell suspensions were injected into the left or right abdominal mammary gland (MG #3 or MG #6); carprofen (50 mg/ml) was applied as analgesic; the skin was closed by interrupted sutures after implantations.

### Immune histochemistry

Histopathology and analysis of transgene expression were essentially as already described [37]. In brief, mouse mammary tissues were fixed with 4% formaldehyde containing 1% acetic acid and embedded in paraffin. De-paraffinated sections were stained with hematoxylin and eosin. Immunostaining of SV40 T-Ag was performed on paraffin sections using a triple-step immune enzymatic method. The sections were reacted before antibody incubation with a commercial 'target unmasking fluid' (Dianova) in a microwave oven. Subsequently, sections were incubated overnight at 48°C with a 1 : 10,000 dilution of the polyclonal rabbit antiserum R15 against T-Ag [38]; in some cases the commercially available polyclonal goat antibody against PD-L1 (R&D Systems) or the mAb against CD3 (BD Biosciences) in dilutions of 1 : 1000 were applied according to the supplier's recommendations. Specifically bound primary antibody was in the case of anti T-Ag, detected using biotinylated anti-rabbit IgG and phosphatase-conjugated streptavidin from a commercial kit (Super Sensitive Detection System, Biogenex). Phosphatase enzyme activity was revealed with naphthol AS-BI phosphate in combination with hexazotized new fuchsine (Merck). For the primary antibodies against PD-L1 and CD3 mouse anti-goat as well as anti-rabbit peroxidase-conjugated antibodies were used, incubated with Histofine Simple Stain Mouse Max PO anti-goat or anti-rabbit (Nichirei, Amsterdam, NL), and detected by 3,3'-diamino benzidine chromogene; possible endogenous peroxidase activities in granulocytes, mast cells, and erythrocytes were blocked by pre-incubation with 30 % H_2_O_2_ solution in phosphate buffered saline. Naïve rabbit serum served as control. Sections were slightly counterstained with hemalum. All photographs were taken by the Zeiss Axioplan2 imaging microscopic equipment with the camera ProgRes C12plus of Jenoptic using the Software ProgRes CapturePro 2.9.0.1.gy.

### Measurement of T-Ag

T-Ag protein and mRNA contents were measured by enzyme-linked immuno-sorbent assay (ELISA) and quantitative real-time polymerase chain reaction (qRT-PCR), respectively, as already described [3]. In short: the protein content was calculated using the Bio-Rad protein assay with the Bradford Reagent [39]. Amounts of T-Ag were determined by ELISA, where aliquots of the samples were adsorbed onto MaxiSorp Immunoplates (Nunc) for 2 h at room temperature, and viral antigen was detected with rabbit anti T-Ag antiserum R15 [38], followed by horseradish peroxidase-labeled goat anti-rabbit immunoglobulins (Medac). T-Ag mRNA was quantitated after extraction of RNA from frozen tissue samples using the innuPrep RNA Mini Kit (Analytic Jena, Germany) and reverse transcription with the High Capacity cDNA Reverse Transcription Kit (Applied Biosystems, Foster City, CA). In general 1 μg of purified RNA was used for the synthesis of cDNA. qRT-PCR was performed with the Power SYBR Green PCR Mastermix in an ABI 7500 Fast thermal cycler (Applied Biosystems). Per 10 μl mix 5 ng of cDNAs and the primer pairs SV40LTag-Q1 (sense: TCCTGGCTGTCTTCATCATC) and SV40LTag-Q2 (antisense: AGAAAGGTTCGACGCTGACAC) in concentrations of 100 nM were used.

## SUPPLEMENTARY FIGURES AND TABLES



## Abbreviations

B_reg_ cells: regulatory B cells
CD: cluster of differentiation
CTL: cytotoxic T lymphocyte
DMEM: Dulbecco's modified Eagle's medium
ELISA: enzyme-linked immunosorbent assay
EMT: epithelial to mesenchymal transition
FCS: fetal calf serum
Gy: Gray
ip: intraperitoneal
iv: intravenous
LCMV: lymphocytic choriomeningitis virus
mAb: monoclonal antibody
MIN: mouse intraepithelial neoplasia
NK cells: natural killer cells
NP: nucleoprotein of LCMV
NP8 mouse: WAP-T_NP_ mouse
PD1: programmed death-1 protein
PD-L1: ligand of PD1
PFU: plaque-forming units
pp: post partum
pw: post weaning
qRT-PCR: quantitative real-time polymerase chain reaction
SV40: Simian virus 40
T1 mouse: WAP-T mouse
T-Ag: T-antigen of SV40
T-Ag_NP_: chimeric T-Ag/NP protein
T_reg_ cells: regulatory T cells
WAP: whey acidic protein
