# Supplementary material for: Immunotherapy of WAP-TNP mice with early stage mammary gland tumors
Source: Oncotarget. 2017 Jun 29;8(40):67790–804. doi: 10.18632/oncotarget.18850 (PMC5620212; doi:10.18632/oncotarget.18850)
Supplement: Supplementary file 1 [file oncotarget-08-67790-s001.pdf]

Immunotherapy of WAP-T_{NP} mice with early stage mammary gland tumors

SUPPLEMENTARY MATERIALS

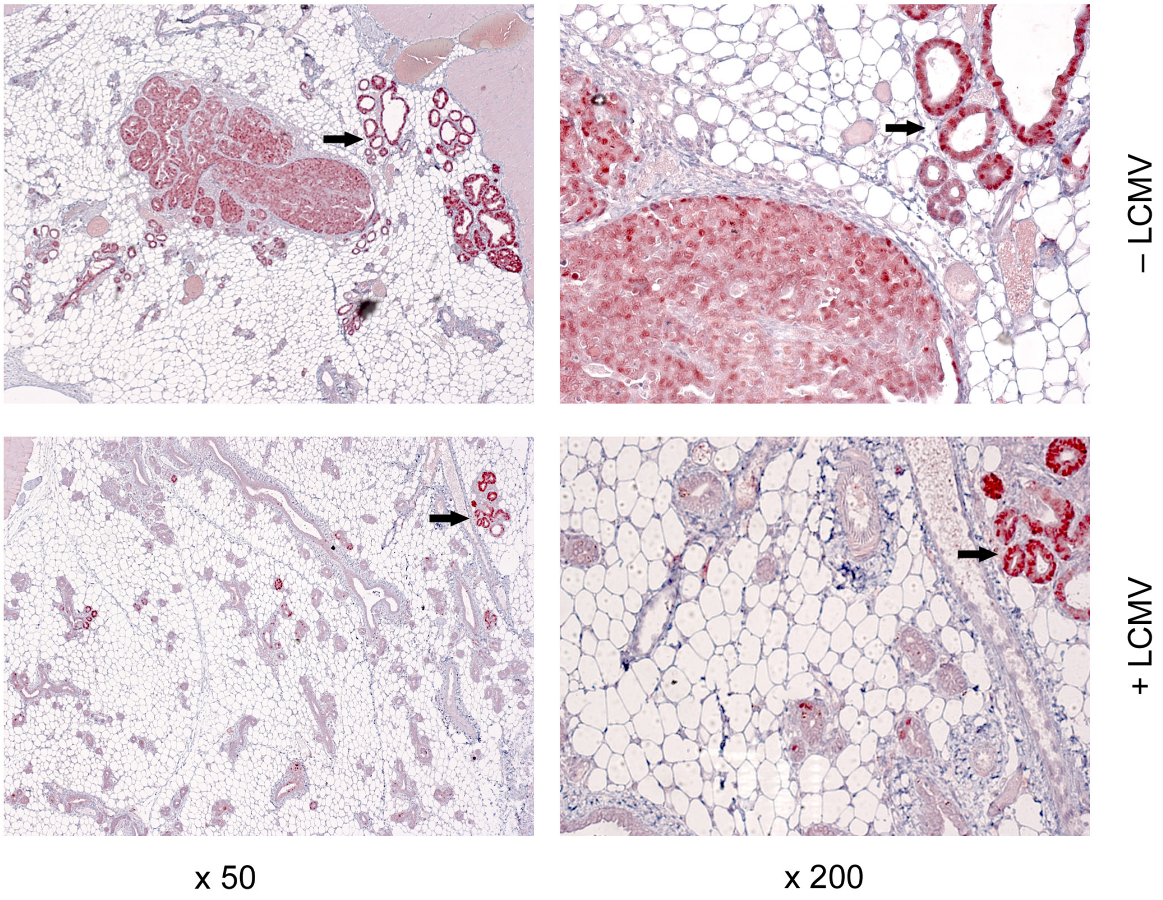

Supplementary Figure 1: Minimal amounts of T-Ag expressing cells (here shown at different magnifications) in NP8 mice infected 7 days pp and analysed on days 200 pw (lower panels) in contrast to untreated controls (upper panels). The arrows point at the same area on the left and right pictures.

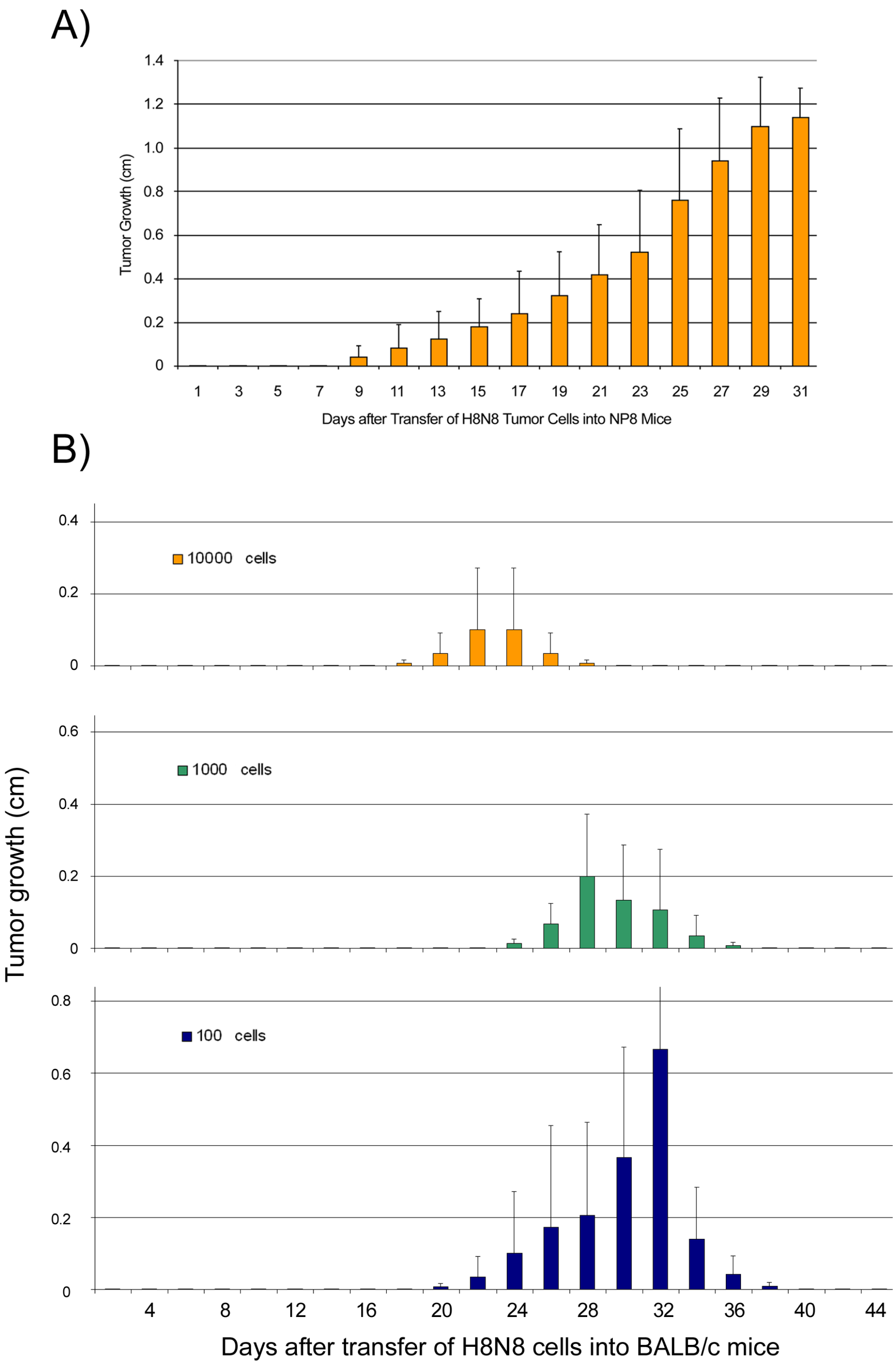

Supplementary Figure 2: Growth of tumors with 10^5 H8N8 cells in NP8 mice **(A)** and 10^2, 10^3, or 10^4 H8N8 cells, respectively, in BALB/c mice **(B)**; with 10^5 H8N8 cells no tumor growth could be measured in BALB/c mice.

Supplementary Table 1: Different curative treatments of NP8 mice via adoptive transfer: presentation of single experiments

Donor mice (wtBALB/c mice)		Acceptor mice (NP8 tumor mice)[a]	
**Transfer of CTLs specific for**	**Day of transfer (pw)**	**Treatment**	**Tumor size 1.5 cm, day pw [mean]**
LCMV	21	None	152 Δ + 45
	21		173 Δ + 64
	28		178 Δ + 71
	35		173 Δ + 64
	50		223 Δ +116 [180]
LCMV	21	+ 4 Gy	183 Δ + 74
	21		195 Δ + 88
	28		188 Δ + 81
	35		205 Δ + 98
	40		210 Δ +103 [196]
H8N8 cells	22	None	75 Δ - 32
	28		115 Δ + 8
	28		120 Δ + 13
	35		125 Δ + 18
	40		110 Δ + 3 [109]
H8N8 cells	21	+ 4 Gy	> 240
	22		> 240
	28		> 240
	28		> 240
	40		> 240 [> 240]
H8N8 cells	42	+ anti PD-L1	126 Δ + 19
	42		164 Δ + 57
	48		151 Δ + 44
	49		153 Δ + 46
	51		170 Δ + 63 [153]
H8N8 cells	42	+ anti PD1	124 Δ + 17
	49		102 Δ +/-
	49		166 Δ + 59
	49		169 Δ + 62
	92		158 Δ + 51 [144]

[a] Untreated NP8 tumor mice (n = 20) [mean of tumor sizes of 1.5 cm at day 107 pw]; Δ in individual experiments refers to the difference between the mean in untreated mice; day of treatment of tumor mice was 1 day before transfer of immune cells.

Supplementary Table 2: Tumor growth in mice after transplantation of H8N8 tumor cells

Cell line	BALB/c	NP8 (uninduced)	T1 (uninduced)
H8N8	0/5[a]	5/5	3/5
H8N8(Arm)	0/5	0/5	0/5

[a] Tumor bearing mice versus total number of mice.
